# Stapedotomy for Pediatric Middle Ear Anomalies With Facial Nerve Bifurcation: A Case Report

**DOI:** 10.7759/cureus.65391

**Published:** 2024-07-25

**Authors:** Yuki Miura, Masao Noda, Ryota Koshu, Makoto Ito

**Affiliations:** 1 Otolaryngology - Head and Neck Surgery, Jichi Medical University, Shimotsuke, JPN

**Keywords:** pediatric surgery, congenital malformation, ossicular chain reconstruction, stapedotomy, facial nerve, facial nerve bifurcation

## Abstract

The intricate distribution of the facial nerve within the temporal bone is crucial in otological surgery. Anomalous facial nerve pathways are occasionally observed in middle ear malformations, although intra-tympanic bifurcation of the facial nerve is rare. When managing ossicular malformations with atypical facial nerve trajectories, hearing reconstruction should be prioritized based on the trajectory pattern and presence of the oval window. In this case, stapes surgery was performed due to facial nerve bifurcation within the tympanic cavity. In this case report, a 15-year-old female underwent stapes surgery due to gradually worsening conductive hearing loss. She was monitored at another hospital because of left-sided hearing loss at birth screening using automated auditory brainstem response. Her left ear initially had mild hearing loss, while her right ear remained within normal limits. However, her hearing deteriorated progressively, leading to significant daily challenges by age seven, prompting referral to our hospital. Intraoperatively, findings included defects in the incus-long process and stapes head, along with facial nerve bifurcation around the oval window, and the stapes footplate had poor flexibility. Stapedotomy was performed cautiously to preserve the facial nerve, utilizing a Teflon piston wire for sound transmission reconstruction. Postoperatively, the patient experienced no complications or facial nerve palsy, with hearing improving to 28.8 dB. Understanding the precise pathophysiology of middle ear anomalies is crucial for selecting appropriate surgical approaches. Even though the anomalies could not be evaluated prior to surgery, surgeons must carefully consider the risk of facial nerve injury and choose the optimal technique and reconstruction method tailored to each case, as predicting outcomes solely from preoperative evaluations can be challenging.

## Introduction

In middle ear surgery for conductive hearing loss, such as middle ear malformations and cholesteatoma, protecting and maintaining facial nerve function, along with preserving hearing, are crucial for the patient's postoperative quality of life. The facial nerve is a complex anatomical structure that extends from the inner ear canal into the temporal bone and the tympanic cavity and controls various functions, including facial movement, taste, and salivation. Its intricate arrangement with multiple branches significantly impacts otological surgery procedures. An abnormal trajectory, especially within the operative field, increases the risk of facial nerve injury during surgery [1,2].

Facial nerve abnormalities are rare, with several variations reported in frequency, ranging from the temporal bone to the mastoid portion [3-5]. The developmental process of the middle ear should consider the abnormal structure of the facial nerve, especially in cases of middle ear malformations, as the facial nerve’s trajectory pattern may influence the choice of the sound transmission reconstruction method [1,2,6].

However, facial nerve bifurcation in the tympanic cavity is rare, and there is no established method for hearing reconstruction in such cases. In this case, a stapedotomy was performed for pediatric middle ear anomalies with a facial nerve bifurcation. Preoperative imaging evaluation was insufficient for making a definitive judgment; therefore, the choice of hearing reconstruction was made intraoperatively. This case highlights the importance of management and surgical approaches for pediatric middle ear anomalies with facial nerve abnormalities.

## Case presentation

A 15-year-old girl presented with hearing loss in her left ear. Automated auditory brainstem response (AABR) screening at birth indicated left-sided hearing loss. Initially monitored for mild left-sided hearing impairment and normal hearing in the right ear at another medical facility, but her left hearing progressively worsened. At age seven, she sought care at our clinic due to significant interference with daily activities by her hearing loss.

**Figure 1:**
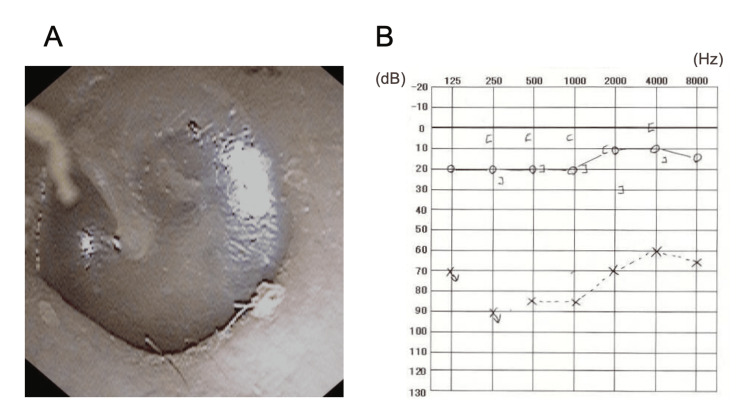
Physical findings of the patient at our department. A: Ear endoscopy shows no perforation or inflammation of the tympanic membrane. B: Pure-tone audiogram demonstrates severe left ear hearing loss with an air-bone gap.

Physical examination revealed no auricular malformations or facial nerve palsy, and the tympanic membrane exhibited no perforations or signs of inflammation (Figure 1A). Pure tone audiometry indicated severe hearing loss on the left side at 81.25 dB using the four-frequency average method, with an air-bone gap of approximately 50 dB before surgery (Figure 1B).

**Figure 2:**
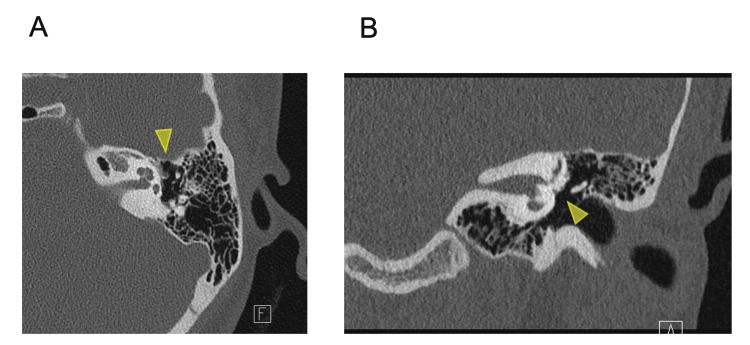
CT scan findings of the patient. CT-scan (A: horizontal section, B: coronal section) showing suspected middle ear anomalies with potential incus-stapes joint deficiency (triangle).

Computed tomography (CT) imaging revealed a defect in the long process of the incus and head of the stapes, suggesting the possible absence of the incus-stapes joint (Figure 2). In addition, incus adhesion on the lateral side of the tympanic cavity was suspected. No obvious inner ear malformations or abnormal facial nerve trajectories were observed.

Based on these findings, conductive hearing loss due to left middle ear abnormalities was suspected. After consultation with the patient and family, although there had been no history of recurring otitis media until then, considering the Eustachian tube’s ventilation capacity and mastoid development, initial conservative management was pursued. However, at age 15, the patient opted for surgery, leading to an exploratory tympanotomy to improve hearing, which examined the tympanic cavity and performed tympanoplasty or stapedotomy, if possible, depending on the conditions.

Surgical procedure

**Figure 3:**
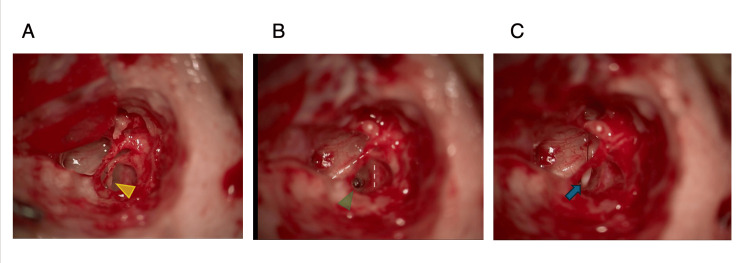
Surgical view of the patient. A: Absence of the incus-long process and stapes head, with bony adherence of the lateral tympanic cavity wall and incus (yellow triangle). B: Skeeter drill opening the stapes footplate (green triangle) between the facial nerves (white dashed line). C: Ossicular chain repair with 5.75 mm Teflon wire piston with a Malleus attachment (blue triangle).

Under general anesthesia, an exploratory tympanotomy was performed. A posterior superior auricular incision was made, followed by access to the tympanic cavity (Figure 3). The absence of the long process of the incus and head of the stapes, along with the bony adherence of the lateral wall of the tympanic cavity and incus, was noted (Figure 3A).

The facial nerve bifurcated near the stapes footplates, with poor flexibility observed in the stapes footplate. With a careful confirmation of the facial nerve trajectory using a nerve integrity monitor (NIM), a stapedotomy was performed by opening the stapes footplate using a skeeter drill with a 0.8mm bar. Ossicular chain repair utilized a 5.75 mm Teflon wire piston with a Malleus attachment (Figure 3B, 3C).

Postoperative course

**Figure 4:**
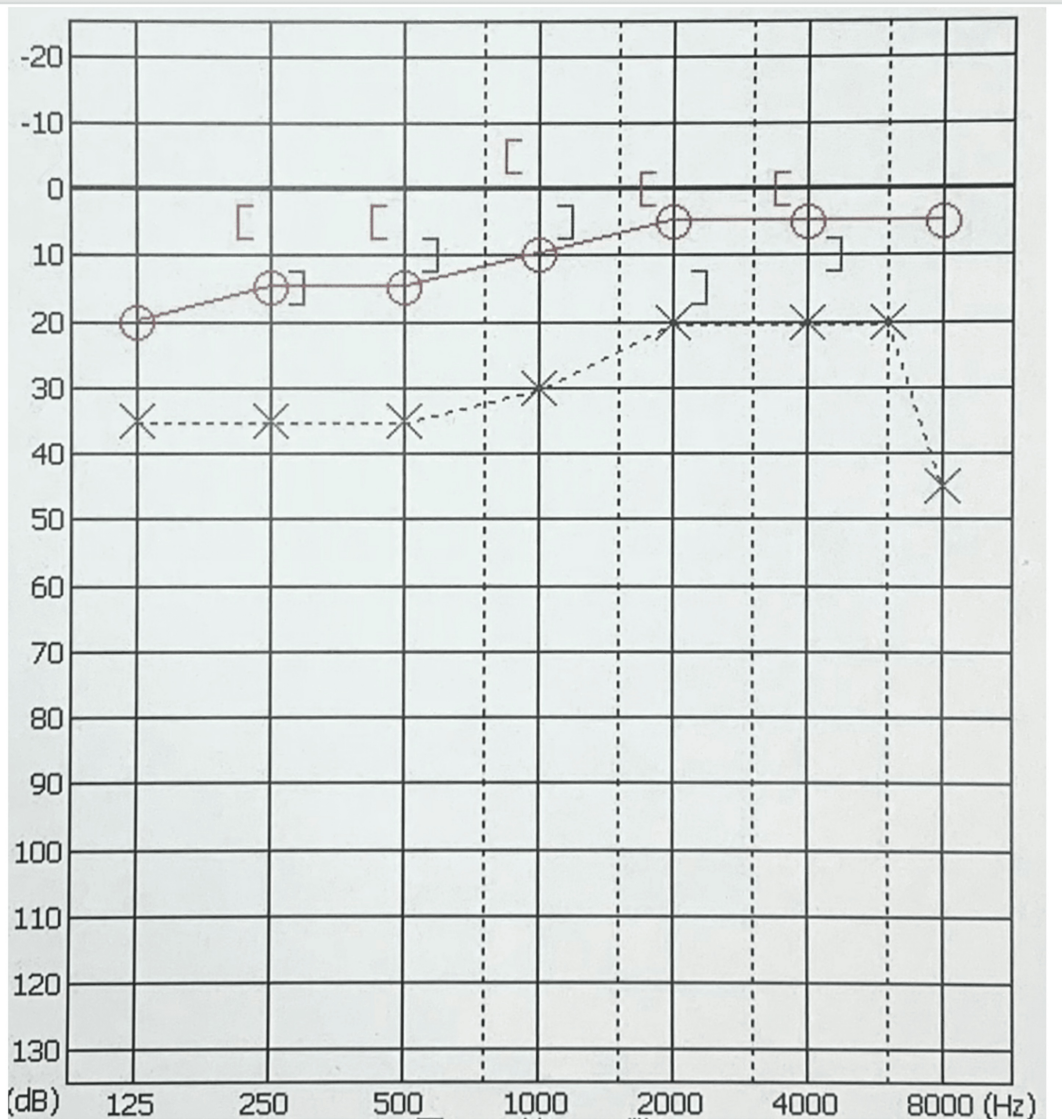
Postoperative pure tone audiogram of the patient.

The patient’s recovery was uneventful, and discharge occurred on the fifth postoperative day. At the 60-day follow-up after surgery, the hearing had improved by more than 40 dB from pre-surgery levels, with no abnormalities in the facial nerve function (Figure 4). Four months have passed since the operation, with no particular problems, such as worsening of hearing or facial paralysis.

## Discussion

We present a case of congenital middle ear malformation with an abnormal facial nerve course within the tympanic cavity, treated successfully with stapes surgery. Intraoperatively, we identified and addressed the facial nerve anomaly, achieving favorable postoperative hearing outcomes without facial nerve palsy.

Embryologically, the facial nerve is closely associated with the auricle, with development beginning at the appearance of nerve primordium at four weeks of gestation [7,8]. The development of the facial nerve proceeds with the formation of the geniculate ganglion in the second half of the fifth week, following a linear course along the second pharyngeal arch ventrally until the second half of the fifth week. Conversely, ossicle development starts in the fourth week of embryonic growth, with the stapes forming from Reichert's cartilage derived from the second pharyngeal arch of the Malleus and Incus long process. By the first half of the sixth week, with the emergence of the stapes protuberans, the nerves are displaced posteriorly to form vertical and horizontal segments, completing the formation of nerve pathways by the second half of the eighth week [7,8]. Therefore, abnormalities involving structures like the malleus, incus long process, and stapes, derived from the second pharyngeal arch as seen in this case, often coincide with facial nerve trajectory abnormalities. Jin et al. reported facial nerve abnormalities in 32.03% (82/256 ears) of cases with middle ear malformations, emphasizing the importance of preoperative evaluation and surgical strategies in patients with oro-facial malformations, considering potential accompanying facial nerve trajectory abnormalities [1].

Advancements in diagnostic imaging techniques have enhanced the accuracy of preoperative assessment of middle ear lesions. High-resolution thin-slice CT scans are now standard for evaluating temporal bone structures, crucial for assessing ossicular abnormalities, their relationship with the facial nerve, and planning safe ear surgeries [9,10]. In addition, three-dimensional CT (3DCT) is valuable in visualizing temporal bone structures, such as the ossicles, facial nerve, and cochlea, in a comprehensible form, aiding surgical planning by providing detailed spatial relationships within the temporal bone postoperatively [11,12]. The 3D image of the temporal bone can be arbitrarily enlarged, rotated, or deleted from the surrounding bone to expose the structures of the middle and inner ears, allowing the surgeon to visually grasp the positional relationship between the normal and abnormal structures within the temporal bone before surgery, and we also previously reported the usefulness of this imaging technique in cochlear implantation [13,14]. Ultra-high-resolution CT (UHRCT) further improves the depiction of microstructures within the temporal bone, including the tensor tympani, facial nerve trajectory, and ligaments in the tympanic cavity, offering enhanced diagnostic capabilities over conventional MDCT [15-18].

Despite these imaging advances, the diverse patterns of middle ear malformations pose challenges in completely evaluating sclerotic lesions and facial nerve trajectory abnormalities preoperatively. Discrepancies between HRCT findings and intraoperative observations underscore the critical role of surgical decision-making during the procedure [3,5].

In our case, facial nerve bifurcation in the tympanic cavity was rare, occurring in only 1.13% of 887 patients who underwent stapes surgery, as reported by Fang et al., where all 10 cases exhibited bifurcation at the horizontal segment of the facial nerve [5]. Jin et al. categorized morphological abnormalities of intratympanic facial nerve courses, noting that most cases involve the nerve anteriorly or inferiorly to the oval window, partially or completely covering it (60.98%). Cases with facial nerve branching around the oval window, the same category as our case, are infrequent (3.66%), highlighting the critical importance of meticulous facial nerve handling for optimal hearing outcomes [1].

Surgical management of facial nerve trajectory abnormalities necessitates tailored techniques. In our case, despite facial nerve bifurcation, the not-close oval window allowed for stapedotomy and hearing reconstruction using a piston wire. However, risks of facial nerve palsy from nerve compression or damage must be considered, particularly if identifying the stapes base plate or oval window proves challenging. Regarding the positional relationship between the facial nerve and the stapes/oval window, if the facial nerve runs away from the stapes footplate/oval window, bone cements or piston wire can be used for ossicular reconstruction, but if the nerve runs over it, a different approach is needed.

Jahrsdoerfer et al. suggested removing facial nerve involving bone surrounded and upward displacement from the oval window to access the stapes footplate, highlighting alternatives in cases of facial nerve displacement [6]. Conversely, reconstruction challenges arise with closed or absent oval windows, necessitating fenestration and piston implantation for sound transmission contingent upon facial nerve position and remaining ossicle development [2].

Recent advancements include using a cochlear implant like the VSB (Vibrant Soundbridge®) or a bone-conducting implant like the BB (BONEBRIDGE®), offering viable alternatives in cases where conventional reconstruction proves challenging [1,19-20].

## Conclusions

In this case, stapedotomy addressed an incus-long process and head of stapes defect alongside facial nerve bifurcation. Given the embryological proximity of the auricle and facial nerve course, meticulous preoperative planning for middle ear malformations is crucial.

The final surgical approach hinges on intraoperative findings. Factors such as nerve trajectory, presence of oval window closure, surgeon expertise, and risk of facial nerve injury delicate the chosen reconstruction method. In this instance, reconstruction proceeded smoothly, yielding excellent postoperative hearing outcomes. However, in select cases, opting against reconstruction remains a viable consideration.
